# Cluster-Assembled Zirconia Substrates Accelerate the Osteogenic Differentiation of Bone Marrow Mesenchymal Stem Cells

**DOI:** 10.3390/nano13050801

**Published:** 2023-02-22

**Authors:** Sara Castiglioni, Laura Locatelli, Alessandra Cazzaniga, Francesca Maria Orecchio, Tommaso Santaniello, Claudio Piazzoni, Lionel Bureau, Francesca Borghi, Paolo Milani, Jeanette A. Maier

**Affiliations:** 1 Department of Biomedical and Clinical Sciences, Università di Milano, 20157 Milano, Italy; 2 Department of Physics and Interdisciplinary Centre for Nanostructured Materials and Interfaces (C.I.Ma.I.Na.[M1]), University of Milan, Via Giovanni Celoria, 16, 20133 Milan, Italy; 3 Laboratoire Interdisciplinaire de Physique (LIPhy), Université Grenoble Alpes, CNRS, F-38000 Grenoble, France

**Keywords:** nanostructured zirconia surfaces, bMSC, osteogenic differentiation

## Abstract

Due to their high mechanical strength and good biocompatibility, nanostructured zirconia surfaces (ns-ZrOx) are widely used for bio-applications. Through supersonic cluster beam deposition, we produced ZrOx films with controllable roughness at the nanoscale, mimicking the morphological and topographical properties of the extracellular matrix. We show that a 20 nm ns-ZrOx surface accelerates the osteogenic differentiation of human bone marrow-derived MSCs (bMSCs) by increasing the deposition of calcium in the extracellular matrix and upregulating some osteogenic differentiation markers. bMSCs seeded on 20 nm ns-ZrOx show randomly oriented actin fibers, changes in nuclear morphology, and a reduction in mitochondrial transmembrane potential when compared to the cells cultured on flat zirconia (flat-ZrO_2_) substrates and glass coverslips used as controls. Additionally, an increase in ROS, known to promote osteogenesis, was detected after 24 h of culture on 20 nm ns-ZrOx. All the modifications induced by the ns-ZrOx surface are rescued after the first hours of culture. We propose that ns-ZrOx-induced cytoskeletal remodeling transmits signals generated by the extracellular environment to the nucleus, with the consequent modulation of the expression of genes controlling cell fate.

## 1. Introduction

The regulation of cell function is mediated by the integration of a set of biochemical and mechanical signals derived from the surrounding cells and the extracellular matrix (ECM). Cells perceive mechanical stimuli by using mechanosensitive molecules at the cell membrane, such as integrins, stretch-activated ion channels, G protein coupled-receptors, and growth factor receptors [1], which transduce signals from the ECM to the inner part of the cell [2]. Remodeling of the cytoskeleton occurs and results in the interaction of actin fibers and microtubules with the nucleus, thus affecting DNA replication, transcription, and gene expression [3,4]. Of note, the nanostructure and composition of the ECM is crucial in modulating the fate commitment of mesenchymal stem cells (MSCs), multipotent cells that can differentiate into osteoblasts, chondrocytes, and adipocytes [5]. Mechanical stimuli trigger the rearrangement of their cytoskeleton and the modification of cell shape, key regulatory events in MSC differentiation [3]. Indeed, it is reported that a spread morphology favors osteogenesis, while a rounded morphology induces adipogenesis [3]. Alterations to the cytoskeleton also involve microtubules, which are important for mitochondrial transport within the cell. Consequently, ECM stiffness and nanostructure can influence mitochondrial morphology and function [6], events required for MSC differentiation. In particular, differentiation activates mitochondria, and the switch from glycolysis to oxidative phosphorylation is essential for the osteogenic differentiation of MSCs [7]. In addition, a decrease in mitochondrial membrane potential has been reported during the differentiation of MSCs [8].

This knowledge opened new perspectives in material science and its application for orthopedic implants. Indeed, even though the bone has a certain regenerative capacity, severe bone defects due to trauma, osteoporosis, cancer, and genetic diseases have become a social problem with a significant impact on health systems. This is the reason why the demand for novel osteoinductive bone biomaterials is steadily increasing [9,10]. These materials should support the adhesion, growth, and osteogenic differentiation of MSCs. Osteoblasts will then secrete collagenous and non-collagenous proteins and promote calcification [11]. Since the topographical structure of bone materials can affect the osteogenic differentiation of MSCs [12], we analyzed the behavior of human MSCs isolated from the bone marrow (bMSCs) seeded on nanostructured matrices. We have chosen to use bMSCs because they are the most commonly utilized stem cell type in clinical trials. We have used the bottom-up nanofabrication method supersonic cluster beam deposition (SCBD) to produce nanostructured surfaces with a reproducible nanoscale roughness parameter, thereby realizing topographies that mimic ECM nano-features [13]. In particular, we produced biocompatible substrates made using zirconia (ZrO_2_) of different roughness (10, 15, and 20 nm). Zirconia is a bioceramic material which is highly biocompatible, is chemically stable, and shows high mechanical strength [14].

A previous study demonstrated in pancreatic β-cells that integrins sense the nanotopography of zirconia and, through actin fibers, the signal then reaches the nucleus, activating a specific transcriptional program which preserves β-cell differentiation and function [15,16]. These nanostructured matrices also promoted neurogenic events when PC12 neuron-like cells and rat hippocampal neurons were used [17].

The objective of this study was to investigate how nanoscale surface topography influences the differentiation of human bMSCs. Understanding how bMSCs respond to mechanical signals could have fundamental implications for tissue engineering and regenerative medicine.

## 2. Materials and Methods

### 2.1. Substrates Preparation

Nanostructured zirconia films with controlled and reproducible nanoscale morphology were produced through supersonic cluster beam deposition (SCBD) using a deposition apparatus equipped with a pulsed microplasma cluster source (PMCS), as described in detail by Piseri et al. and Schulte et al. [18,19]. In the PMCS, an argon plasma jet ignited by a pulsed electric discharge ablates a zirconium rod. Zr atoms and ions sputtered from the target thermalize with the argon and traces of oxygen present in the condensation chamber and aggregate to form ZrOx clusters. The mixture of clusters and inert gas then expands into a vacuum chamber, through a nozzle, to form a seeded supersonic beam. The clusters carried by the seeded supersonic beam are collected on a substrate intersecting the beam’s trajectory (deposition rate of about 0.5–2.5 nm/min) and placed in a second vacuum chamber, thus forming a cluster-assembled film. Further oxidation of ZrOx clusters takes place upon exposure to ambient atmosphere, thus forming a ZrOx film. Three different batches of cluster-assembled ZrO_2_ films (called ns-ZrOx hereafter) with a roughness (Rq) of 10, 15, or 20 nm were produced on round glass coverslips (Ø13 mm). As a reference, we also produced flat ZrO_2_ films (Rq = 0.4 nm) through electron beam evaporation of a solid Zr target (flat-ZrO_2_). For the experiments, the glass and zirconia surfaces were sterilized with UV light for 30 min before the cells were seeded.

### 2.2. Atomic Force Microscopy Characterization

**Figure 1:**
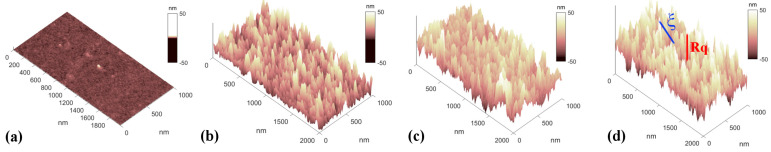
Four 3D AFM images of the glass substrate (**a**) and of the nanostructured zirconia surfaces with increasing roughness ((**b**–**d**), from 10 nm to 20 nm). The morphological parameters calculated by the height maps, i.e., surface roughness (Rq) and correlation length (ξ), have been schematically indicated by the red and blue lines.

The morphology of zirconia samples was characterized by an Atomic Force Microscopy multimode nanoscope (Bruker). Several micrometers-large topographic images of the zirconia surface were collected by operating the nanoscope in tapping mode in air with scan rates of 1 Hz. Representative AFM images of the glass substrate and of the rough zirconia surfaces are reported in Figure 1, showing a granular rough morphology resulting from the stacking of zirconia clusters a few nanometers large (according to a ballistic deposition model) [20,21]. From the AFM images, two morphological parameters were calculated: root mean square roughness (Rq, schematically drawn by the red line in Figure 1d), which stands for the standard deviation of the heights, and correlation length (ξ, blue line in Figure 1d), which is a statistical measurement of the average peak-to-valley lateral dimension of the largest morphological surface features. These morphological properties depend on the thickness of the film in accordance with simple scaling law [20,22].

Interestingly, a critical ligand spacing between the adhesion sites of integrin clusters has been identified as having an influence on cellular capability to establish focal adhesion sites [23,24] and mechanotransduction events [25]. This thresholding distance (~60–70 nm) is in the range of the median of the asperity separation of the 20 nm rough zirconia surfaces, i.e., the abovementioned correlation length ξ.

### 2.3. Culture of Human bMSCs

Human bone marrow derived MSCs (bMSCs) isolated from a female donor were purchased from CliniSciences (Nanterre, France). Two different strains of cells derived from two donors were utilized and yielded similar results. The cells, used between passage 2 and 6, were cultured at 37 °C and under 5% CO_2_ conditions in Dulbecco’s Modified Eagle’s Medium containing 10% fetal bovine serum and 2 mM glutamine (culture medium, CM). To induce osteogenic differentiation, bMSCs were seeded in 24-well plates in the presence of different substrates (glass, flat-ZrO_2_, ns-ZrOx). Once the cells were confluent, an osteogenic induction cocktail was added to the medium (osteogenic medium, OM). The osteogenic cocktail contained 2 × 10^−8^ M 1α,25-Dihydroxyvitamin D_3_, 10 mM β-glycerolphosphate, and 0.05 mM ascorbic acid. All the reagents for cell culture were from Sigma-Aldrich (St. Louis, MO, USA). To analyze calcium deposition by bMSCs, the cells were rinsed with Phosphate Buffered Saline and then fixed (70% ethanol, 1 h) and stained for 10 min with 2% Alizarin Red S (pH 4.2, Sigma-Aldrich). Alizarin Red S staining was released from the cell matrix through incubation in 10% cetylpyridinium chloride in 10 mM sodium phosphate (pH 7.0) for 15 min, and absorbance was measured at 562 nm using a Varioskan LUX Multimode Microplate Reader (Thermo Fisher Scientific, Waltham, MA, USA). The experiment was repeated three times in triplicate. Images were taken using an optical microscope at 10× magnification.

### 2.4. Real-Time PCR

Total RNA was extracted by the PureLink RNA Mini kit (Thermo Fisher Scientific, Waltham, MA, USA). Single-stranded cDNA was synthesized from 0.3 μg of RNA in a 20 μL final volume using a High-Capacity cDNA Reverse Transcription Kit with RNase inhibitors (Thermo Fisher Scientific, Waltham, MA, USA) according to the manufacturer’s instructions. Real-time PCR was performed on 20 ng of cDNA using TaqMan™ Fast Universal PCR Master Mix (Thermo Fisher Scientific, Waltham, MA, USA) and TaqMan Gene Expression Assays (FAM) (Thermo Fisher Scientific, Waltham, MA, USA). The following primers were used: Runt-related transcription factor *(RUNX)2* (Hs00231692_m1), *Sp7* (Hs01866874_s1), Collagen Type I Alpha 1 chain (*COL1A1*) (Hs00164004_m1), Bone Gamma-Carboxyglutamate Protein (*BGLAP*) (Hs01587814_g1), Secreted Phosphoprotein (*SPP)1* (Hs00959010_m1), and Peroxisome Proliferator-Activated Receptor γ (*PPARγ*) (Hs01115513_m1). The housekeeping gene Glyceraldehyde-3-phosphate Dehydrogenase (*GAPDH)* (Hs99999905_m1) was used as an internal reference gene. The reactions were performed with a CFX96 Real-Time PCR Detection System (Bio-Rad, Hercules, CA, USA). Relative changes in gene expression were analyzed using the 2^−ΔΔCt^ method.

### 2.5. Confocal Microscopy

bMSCs were fixed in phosphate buffered saline containing 4% paraformaldehyde and 2% sucrose (pH 7.6), permeabilized with Triton 0.3%, incubated with anti-cyclophilin F (Invitrogen, Waltham, MA, USA) overnight at 4 °C, and stained with Alexa Fluor 488 secondary antibody (Thermo Fisher Scientific, Waltham, MA, USA). We used tetramethylrhodamine (TRITC)-labeled phalloidin (Sigma-Aldrich, St. Louis, MO, USA) to visualize the cytoskeleton. 4′,6-Diamidine-2′-phenylindole dihydrochloride (DAPI, Sigma-Aldrich, St. Louis, MO, USA) was used to stain the nuclei. Finally, cells were mounted with ProLong Gold Antifade Mountant (Invitrogen, Carlsbad, CA, USA) and images were acquired using a 40× objective in oil through the use of an SP8 Leica confocal microscope.

### 2.6. Nuclei Morphology

Nuclear shape quantification was performed using ImageJ on samples stained with DAPI. Briefly, images taken using the confocal microscope were opened in ImageJ, nuclei were contoured with the “polygon selection” function, and images were then analyzed with the “shape descriptor” function. The major/minor axis value describes the tendency toward a perfect circle (ratio = 1) or an elongated/elliptical shape (ratio > 1).

### 2.7. Actin Alignment Quantification

Images derived from fluorescence microscopy for F-actin on bMSCs were analyzed using the OrientationJ plugin of ImageJ, as described in detail in [26,27], in order to compute an orientation map. An angle theta, which corresponds to the preferred distribution acquired by the cytoskeleton, was attributed to every pixel involved in oriented actin structures and angle distribution (fraction of pixels involved in an oriented structure with a given angle in the range of −90° to +90° around the most represented angle (arbitrarily set to 0°)) was plotted.

### 2.8. Mitochondrial Membrane Polarization

For analysis of mitochondrial membrane polarization, bMSCs were stained with 10 µg/mL JC-1 (5,5′,6,6′-tetrachloro-1,1′,3,3′-tetraethylbenzimidazolylcarbocynanine iodide; Invitrogen, Carlsbad, CA) for 10 min at 37 °C in the dark and washed twice with PBS before analysis. Fluorescence was acquired at λex = 485 nm and λem = 530 nm (green fluorescence),as well as at λex = 535 nm and λem = 590 nm (red fluorescence), and cells were then imaged using a 63× objective in oil through the use of an SP8 Leica confocal microscope. Mitochondrial membrane depolarization can be monitored by changes in the JC-1 red/green fluorescence ratio, where a decreased ratio is indicative of decreased mitochondrial membrane potential. Experiments were performed three times in triplicate ± standard deviation (SD).

### 2.9. Reactive Oxygen Species (ROS) Production Analysis

For the detection of ROS, bMSCs were incubated with 10 µM of 2′-7′-dichlorofluorescein diacetate (DCFDA) (Thermo Fisher Scientific, Waltham, MA, USA) solution for 30 min at 37 °C. DCFDA was deacetylated by cellular esterases to a non-fluorescent compound, which was then oxidized by ROS into the fluorescent molecule 2′,7′-dichlorofluorescein (DCF) (λexc = 495 nm, λemm = 529 nm). Dye fluorescent emission was measured using a Varioskan LUX Multimode Microplate Reader (Thermo Fisher Scientific, Waltham, MA, USA). DCFDA fluorescence was normalized on nuclei after DAPI staining. The results shown are the mean of three independent experiments performed in triplicate ± SD.

### 2.10. Statistical Analysis

The data were analyzed using two-way ANOVA. The *p*-values deriving from multiple comparisons were corrected using the Tukey method. Statistical significance was defined as a *p*-value ≤ 0.05 (* *p* ≤ 0.05; ** *p* ≤ 0.01; *** *p* ≤ 0.001).

## 3. Results

### 3.1. Nanostructured Zirconia Substrates Accelerate bMSC Osteogenic Differentiation

**Figure 2:**
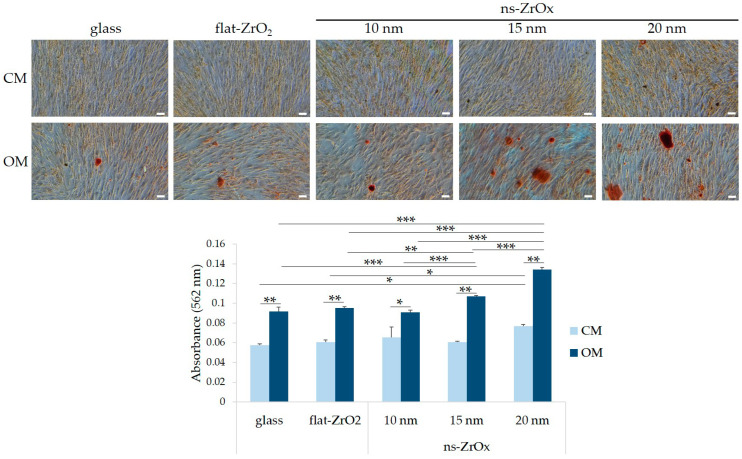
The effects of nanostructured zirconia substrates on the deposition of calcium in the ECM. bMSCs were cultured in CM or OM on ns-ZrOx of different roughness (10, 15, and 20 nm). The flat-ZrO_2_ substrate and glass coverslips (glass) were used as controls. After 15 days, Alizarin Red S staining was performed and photographs were taken at 10× magnification (**upper panel**). Scale bar: 150 µm. After acid extraction, absorbance was measured at 562 nm (**lower panel**). * *p* ≤ 0.05; ** *p* ≤ 0.01; *** *p* ≤ 0.001.

**Figure 3:**
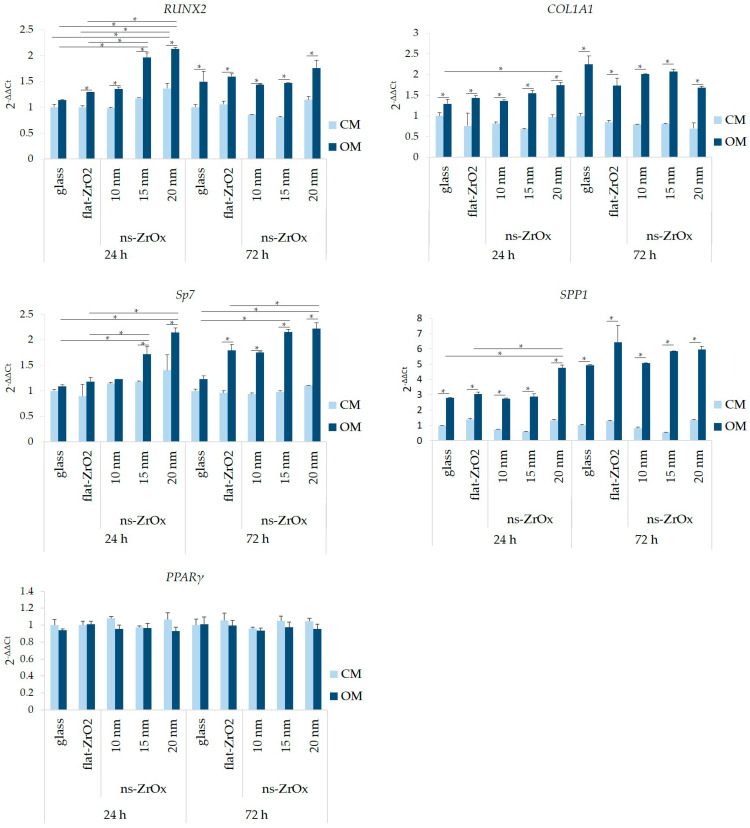
The effects of nanostructured zirconia substrates on bMSC differentiation. RT-PCR was performed on RNA extracted from bMSCs cultured in CM or OM for 24 and 72 h on ns-ZrOx (20 nm), flat-ZrO_2_, and glass using primers designed on *RUNX2*, *COL1A1*, *Sp7*, *SPP1*, and *PPARγ* sequences. * *p* ≤ 0.05.

We analyzed if the presence of nanostructured substrates affects the osteogenic differentiation of bMSCs. Cells were seeded on nanostructured substrates (ns-ZrOx) of different roughness (10, 15, and 20 nm). Flat zirconia (flat-ZrO_2_) substrates and glass coverslips were used as controls. Once confluent, bMSCs were cultured in their culture medium (CM) or in the osteogenic medium (OM). Initially, we investigated the osteogenic differentiation of bMSCs by evaluating the deposition of calcium in the ECM. Figure 2 shows a significant increase in calcium deposits in the cells cultured for 15 days in OM on 15 and 20 nm ns-ZrOx compared to cells cultured on flat-ZrO_2_ and glass. Cells cultured on 10 nm ns-ZrOx behaved in a similar manner to the controls. Interestingly, some calcium deposits were also detected in the cells cultured in CM on 20 nm ns-ZrOx (Figure 2). Through the use of RT-PCR, we analyzed the expression of some osteogenic markers after 24 and 72 h of culture in CM or OM. As expected [28,29], the OM induces the upregulation of all the osteogenic markers considered. Interestingly, after 24 h, we found a significant increase in the transcripts for *RUNX2* and *Sp7* in the cells induced to differentiate on 15 and 20 nm ns-ZrOx when compared to flat-ZrO_2_ and glass (Figure 3). The cells differentiating on 20 nm ns-ZrOx also upregulated *COL1A1* and *SPP1* transcripts. An increase in *RUNX2* and *Sp7*, even if not statistically significant, was also observed in bMSCs cultured on 20 nm ns-ZrOx in CM vs. their controls (Figure 3). No differences were detected in the expression of osteogenic markers after 72 h of cell culture on the different surfaces. We found no modulation of *PPARγ*, the master regulator of adipogenesis, in the experimental conditions tested (Figure 3).

Since 20 nm ns-ZrOx upregulated several osteogenic markers and increased calcium deposition, we decided to perform the following experiments on the 20 nm nanostructure.

### 3.2. The 20 nm ns-ZrOx Substrate Increases ROS Production in bMSCs

**Figure 4:**
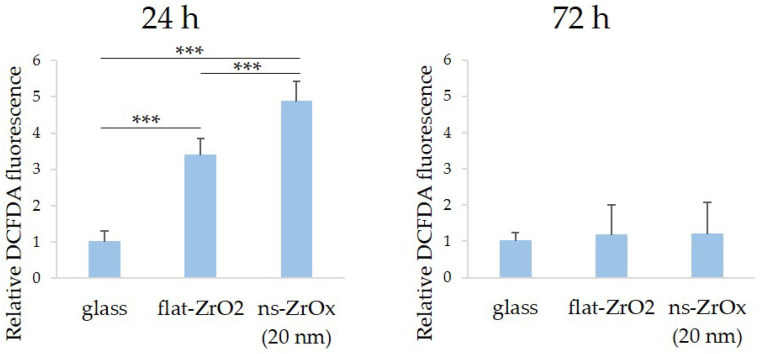
The effect of 20 nm ns-ZrOx substrate on ROS production. bMSCs were cultured on 20 nm ns-ZrOx, flat-ZrO_2_, and glass. After 24 and 72 h, ROS accumulation was measured using a 2′,7′-dichlorofluorescein (DCFDA) assay. DCFDA fluorescence was normalized to DAPI fluorescence and expressed compared to the cells cultured on glass. *** *p* ≤ 0.001.

Since ROS generation is needed for osteogenic differentiation of bMSCs, we analyzed ROS production 24 h after seeding on 20 nm ns-ZrOx and found that flat-ZrO_2_ increases ROS, and this is further accentuated by culture on 20 nm ns-ZrOx (Figure 4). After 72 h of cell culture on the different substrates, ROS reverted to baseline.

### 3.3. The 20 nm ns-ZrOx Substrate Remodels the Actin Cytoskeleton of bMSCs

**Figure 5:**
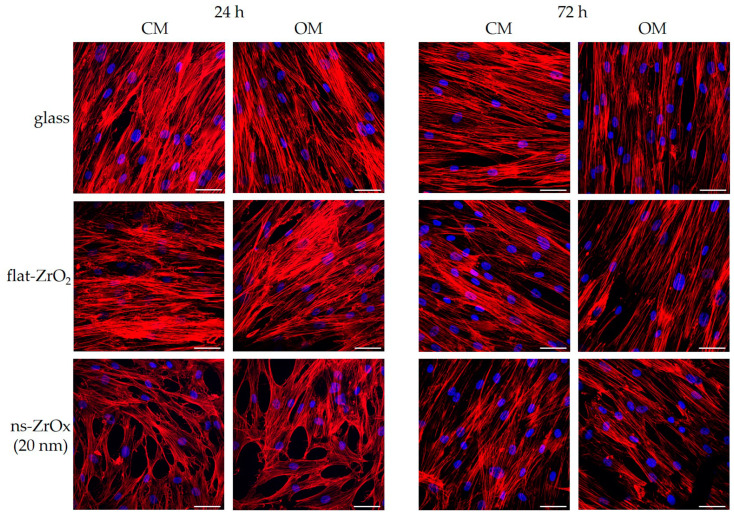
The effect of 20 nm ns-ZrOx substrate on the actin cytoskeleton. bMSCs were cultured in CM or OM on 20 nm ns-ZrOx, flat-ZrO_2_, and glass. After 24 and 72 h, the effects of the substrate were observed through confocal microscopy after TRITC-phalloidin and DAPI staining. Scale bar: 50 µm.

To test the effects of nanostructurated substrate on bMSC morphology, we analyzed the actin cytoskeleton of the cells cultured on 20 nm ns-ZrOx, flat-ZrO_2_, and glass through immunofluorescence. As shown in Figure 5, after 24 h of culture and independently of the medium used, bMSCs seeded on glass and flat-ZrO_2_ appear elongated with parallel, well-organized, and long actin filaments, while the cells on the nanostructured substrate have polygonal shapes with actin fibers oriented in a chaotic manner. These alterations revert after 72 h. 

**Figure 6:**
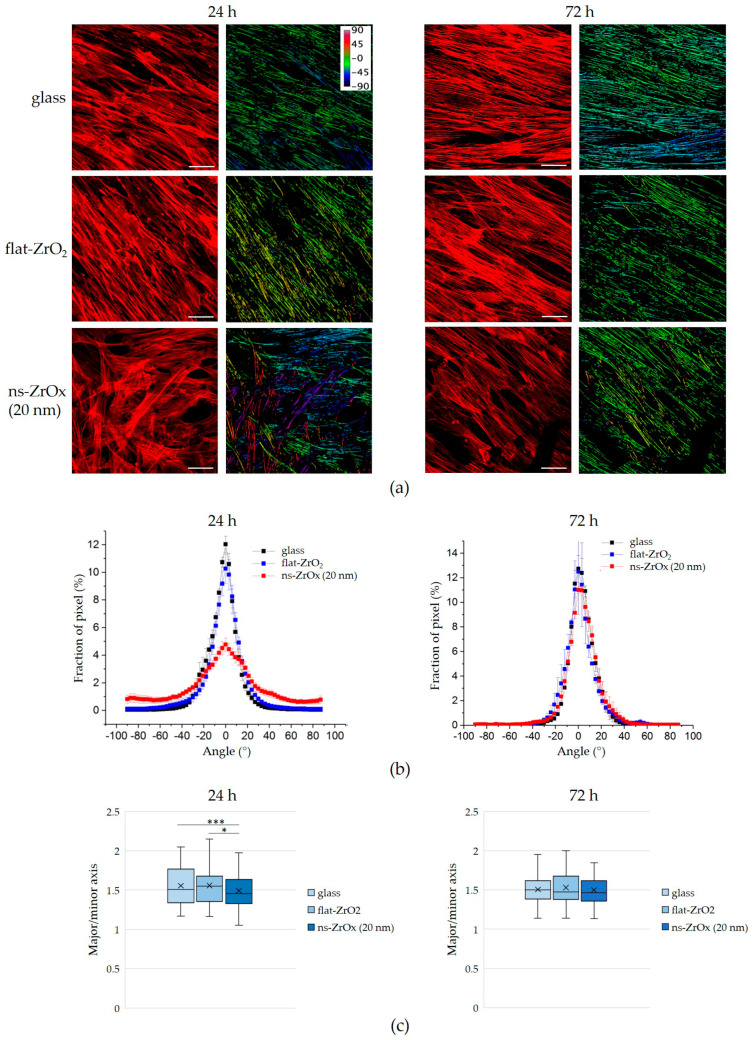
The effect of 20 nm ns-ZrOx on the organization of actin fibers and nuclear architecture. bMSCs were cultured on 20 nm ns-ZrOx, flat-ZrO_2_, and glass for 24 and 72 h. (**a**) The respective orientation analysis of actin fibers was performed using ImageJ. The images in the left panel were obtained via confocal microscopy; the images in the right panel represent the respective orientation analysis obtained using ImageJ. Actin fibers of the same color indicate a well-oriented and organized cytoskeleton. In contrast, actin fibers with different colors indicate the presence of randomly organized fibers. Scale bar: 50 µm. (**b**) The graphs show the angle distribution of actin fibers in the range of −90° to +90° around the most represented angle. (**c**) Analyses of nuclear architecture (major/minor axis). * *p* ≤ 0.05; *** *p* ≤ 0.001.

We then quantified the organization of actin fiber distribution within the cells cultured on the different substrates. An orientation mapping was performed (Figure 6a,b) in which the angle distribution of actin fibers in the range of −90° to +90° around the most represented angle (arbitrarily set to 0°) was computed. After 24 h, the cells cultured on glass and flat-ZrO_2_ had a preferred orientation of actin fibers, which is visible in the distributions as a clear peak, whereas cells on 20 nm ns-ZrOx organized their actin randomly, yielding angle distributions that were broader and less peaked. These data demonstrate a general reorganization of the cell cytoskeleton on ns-ZrOx. After 72 h of cell culture, no morphological differences were detected between cells cultured on the nanostructured substrate and the control substrates, and, in parallel, no differences in the angular distributions of the actin fibers were observed (Figure 6a,b). A similar trend of angular distributions was observed in both CM and OM.

Since reorganization of the actin cytoskeleton might also induce modifications to nuclear morphology, we analyzed the nuclear roundness (major/minor axis) of the cells cultured on the different substrates. DAPI-stained nuclei of the cells cultured for 24 h on 20 ns-ZrOx were rounder than in the controls (Figure 6c). As for the actin filaments, after 72 h of culture, no differences in nuclei morphology are appreciable (Figure 6c).

### 3.4. The 20 nm ns-ZrOx Substrate Induces Mitochondrial Membrane Depolarization

**Figure 7:**
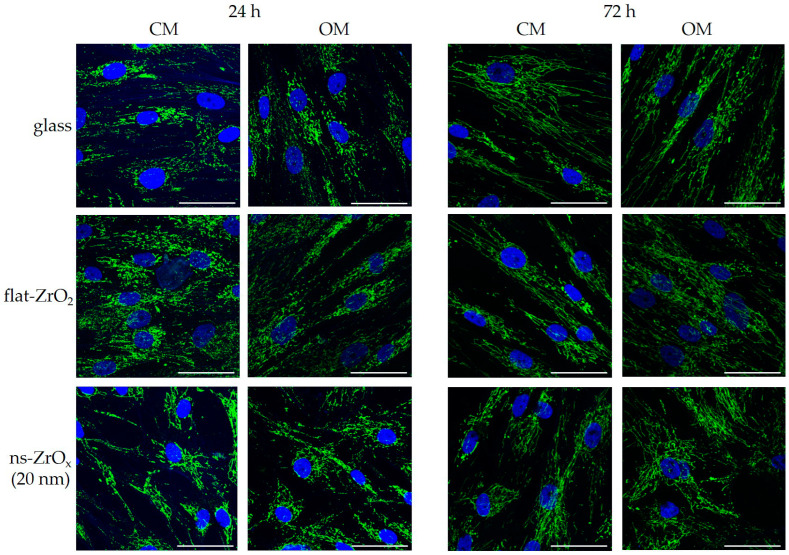
The effect of 20 nm ns-ZrOx on the mitochondrial network. bMSCs were cultured in CM or OM on 20 nm ns-ZrOx, flat-ZrO_2_, and glass. After 24 and 72 h, they were observed via confocal microscopy after cyclophilin F and DAPI staining. Scale bar: 50 µm.

Since mitochondrial dynamics and function are strictly linked to the cytoskeleton [6], we performed some analyses on mitochondria to understand whether the early disorganization of the cytoskeleton was associated with altered mitochondrial remodeling. Mitochondria were stained with antibodies against cyclophilin F and analyzed via confocal microscopy. As shown in Figure 7, all the cells displayed a complex mitochondrial network with elongated mitochondria. Some differences can be appreciated in the distribution of mitochondrial networks in cells cultured on 20 nm ns-ZrOx for 24 h both in CM and OM, probably because of the altered organization of the cytoskeleton (Figure 5).

**Figure 8:**
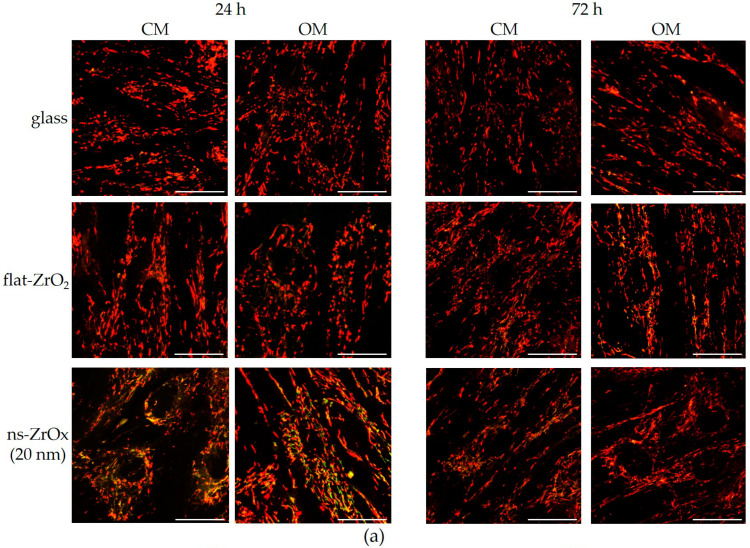

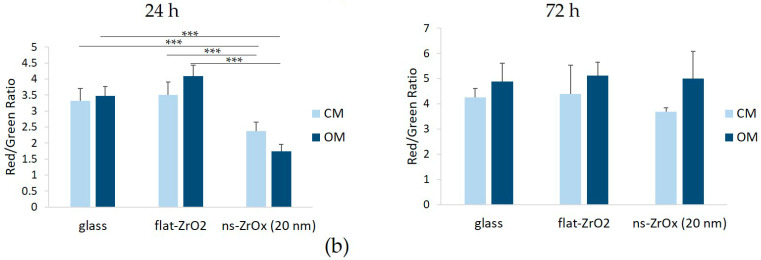
The effect of 20 nm ns-ZrOx on mitochondrial membrane potential. bMSCs were cultured in CM or OM on 20 nm ns-ZrOx, flat-ZrO_2_, and glass. After JC-1 staining, the cells were observed through confocal microscopy (**a**) and the red/green fluorescence ratio was measured (**b**). The decreased ratio is indicative of decreased mitochondrial membrane potential. Scale bar: 30 µm. *** *p* ≤ 0.001.

Using the JC-1 dye, we measured mitochondrial membrane potential and found it to be significantly depolarized in bMSCs cultured for 24 h on 20 nm ns-ZrOx vs. the cells cultured on glass or flat-ZrO_2_, independent of culture conditions (Figure 8). After 72 h of culture, these differences in mitochondrial membrane polarization were not detected any longer (Figure 8).

## 4. Discussion

The bone is a metabolically active tissue that undergoes continuous remodeling throughout life to repair microdamage and modify its architecture to meet changing mechanical needs. This dynamic process is coordinated by the action of osteoclasts and osteoblasts, which are responsible for bone resorption and bone formation, respectively. Osteoblasts derive from bMSCs, multipotent stem cells that, in response to specific stimuli, activate the genetic program leading to osteoblastogenesis. Both biochemical and physical factors are important in directing the fate commitment of bMSCs. Among physical factors, cell shape, external mechanical forces, the ECM, and geometric structures have been implicated in bMSC differentiation [30]. In particular, variations of a few nanometers in surface topography suffice to modulate bMSC behavior, from cell adhesion and spreading to proliferation and differentiation [31].

Here, we analyzed how nanostructured zirconia surfaces influence the osteogenic differentiation of bMSCs. Due to its high mechanical strength and good biocompatibility, ZrOx is widely used for bio-applications, among which orthopedic and dental implants. We produced nanostructured ZrOx films on flat substrates with disordered yet controlled topographic features through SCBD. These films possess controllable roughness at the nanoscale, mimicking the morphological and topographical properties of the ECM. We show that seeding bMSCs on 15 and 20 nm (but not 10 nm) ns-ZrOx in the presence of an osteogenic cocktail accelerates their differentiation, as detected by the increase of the deposition of calcium in the ECM and the upregulation of several osteogenic differentiation markers. In particular, we detected the upregulation of RNA coding for two transcription factors, i.e., *RUNX2*, which is the master switch of osteogenesis [32], and *Sp7*, which is necessary for bone mineralization [33]. In bMSCs seeded on 20 nm ns-ZrOx, we observed the overexpression of *COL1A1*, coding for collagen 1A1 that represents 90% of the organic component of bone matrix [34], and osteopontin (*SPP1*), which is, together with osteocalcin, the most abundant non-collagenous component of bone ECM. Interestingly, the 20 nm ns-ZrOx surface is able to induce the osteogenesis of bMSCs even in the absence of the osteogenic cocktail, as demonstrated by the presence of some calcium deposits and the upregulation of *RUNX2* in the cells cultured on a nanostructured surface in their normal CM. Conversely, the 10 nm ns-ZrOx surface exerts no effect, a result that underlies the relevance of surface roughness in modulating bMSC fate.

Since nanostructured ZrOx surfaces rapidly induce a transient reorganization of the bMSC cytoskeleton with randomly oriented actin fibers, we propose that cytoskeletal remodeling plays a role in accelerating bMSC differentiation. Indeed, beyond maintaining cell shape, the cytoskeleton is central in mechanosensing and mechanosignaling [35]. It also contributes to the transport of molecules within the cell, as well as to the correct subcellular localization and function of various organelles [1], including mitochondria and nuclei [36]. Mitochondria interact with components of the cytoskeleton and utilize cytoskeletal proteins as trails for their movement [37]. Accordingly, in bMSCs seeded on nanostructured ZrOx, the different distribution of mitochondria is likely mediated by the altered orientation of actin fibers. Additionally, the nuclei roundness of bMSCs was increased after 24 h of culture on the nanostructured surface. Changes in nuclear morphology have been described in differentiating embryo stem cells and are related to the modulation of gene expression [38,39]. The remodeling of nuclear size and shape has been reported in the adipogenic differentiation of bMSCs [40], though this issue seems to be overlooked in osteogenic differentiation. Of note, these events are transient, and no differences are detected after 72 h between cells on nanostructured or control surfaces. At the biochemical level, after 24 h of culture on 20 nm ns-ZrOx surfaces, we observed an accumulation of ROS and mildly reduced mitochondrial transmembrane potential, both of which were reversed 72 h later. An adequate amount of ROS is necessary to influence differentiation [41], and, accordingly, a modest increase in ROS accelerates the osteogenic differentiation of bMSCs [42,43]; however, controversial results are available on the interplay between ROS and mitochondrial potential. A mild depolarization of the mitochondria is sufficient to completely inhibit mitochondrial ROS generation [44]. It is also reported, however, that depolarization of the mitochondrial membrane can induce ROS accumulation and, in turn, an increase in ROS could potentiate this depolarization [45]. At the moment, we have no evidence to support whether an increase in ROS is or is not caused by mitochondrial depolarization. Of interest, our results support the findings of Kalmadinov et al. [46], who demonstrated that bMSCs with mildly depolarized mitochondria downregulate senescence-associated markers and upregulate stemness markers, thus indicating a higher regenerative capacity. Additionally, autophagy markers are overexpressed in respect to bMSCs with hyperpolarized mitochondria, and it is known that autophagy contributes to the regulation of bMSC osteogenic differentiation [28]. It is also worth noting that all the effects observed in bMSCs cultured on the 20 nm ns-ZrOx surface are transient. Different kinds of stress—oxidative, mechanical, and metabolic—enhance cell differentiation [47,48,49]. It is feasible that culture on nanostructured surfaces is perceived as a stressful condition, which primes the cells to a phase of readiness to adapt to an unusual environment. We hypothesize that different mechanisms, such as the upregulation of stress proteins and anti-oxidative responses, might be involved in enabling cell adaptation to micro-environmental challenges.

## 5. Conclusions

We propose that signals generated by the 20 nm ns-ZrOx surfaces transiently remodel the cytoskeleton, which transmits the information to the nucleus, thus triggering the rapid activation of the osteogenic program in human bMSCs.

While further experiments are necessary to prove this hypothesis, our results point to zirconia nanostructured surfaces as a promising tool in regenerative medicine.

## Data Availability

The data presented in this study are openly available in Dataverse through the following link: https://dataverse.unimi.it/dataverse/ZirconiaSubstrates (accessed on 31 January 2023).
